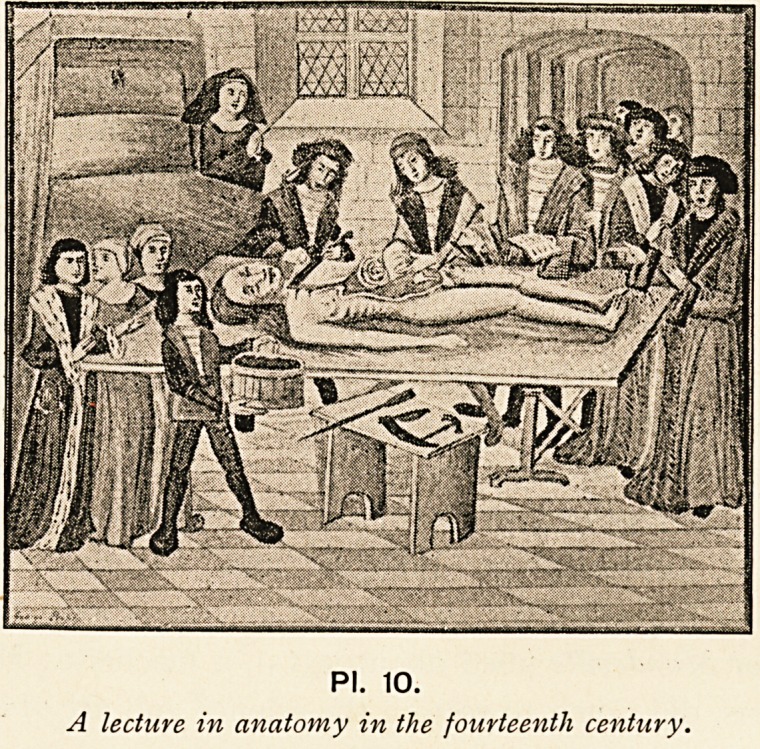# The History and Practice of Surgery in Ancient and Mediæval Times

**Published:** 1909-12

**Authors:** James Swain

**Affiliations:** Professor of Surgery in the University of Bristol; Surgeon to the Bristol Royal Infirmary


"*Scire est nescire, nisi id me*

*Scire alius sciret.*"

DECEMBER, 1909.

INDEX THROUGH

### THE HISTORY AND PRACTICE OF SURGERY IN ANCIENT AND MEDIÆVAL TIMES.

The President's Address, delivered on October 13th, 1909, at the opening of the Thirty-sixth Session of the Bristol Medico-Chirurgical Society.

BY

JAMES SWAIN, M.S., M.D. Lond., F.R.C.S. Eng.,

*Professor of Surgery in the University of Bristol; Surgeon to the Bristol Royal Infirmary.*

"Is there anything whereof it may be said, 'See, this is new'?"

THE progress of modern surgery owes its recent phenomenal success and expansion to the use of anæsthetics and the prevention of sepsis in wounds.

In 1844 the American dentist Horace Wells demonstrated the practicability of using nitrous oxide gas for surgical anæsthesia; within a few years (1846) ether was used for a similar purpose by Mr. Morton, also an American dentist; and Sir James Young Simpson, of Edinburgh, employed chloroform (1847) as a substitute for ether. In 1867 Lord Lister began to publish papers on the antiseptic principle in the practice of surgery, with which his name will always be illustriously associated.

The brilliant achievement of Horace Wells was, however, made possible by the memorable work of Sir Humphry Davy, who discovered the anæsthetic properties of nitrous oxide gas in 1799, when he was carrying out important experiments in the Pneumatic Institution founded by Dr. Thomas Beddoes in Dowry Square, in this city of Bristol. The great genius which Lister displayed in the application of the "germ theory" to the treatment of wounds was similarly dependent upon the work of his predecessors, more particularly that of Pasteur, who demonstrated in 1857 that fermentation and putrefaction were due to micro-organisms, and that of Runge, who discovered carbolic acid in 1834.

Like Lister and Wells, we are the heirs of the ages, and the foundation of surgery is to be sought for in the distant past. This heritage is often forgotten in the feverish haste of modern times, and the glamour of our science and art of to-day is apt to blind our eyes to the obligation we are under to those who worked with so few of the advantages which we ourselves possess.

The evolution of surgery continues to ebb and flow as it always has done. To-day, as before, we have to change our ideas with the progress of knowledge; and therefore, instead of wearying you with the details of what may be considered the latest and best of surgical knowledge, I propose to put before you a brief history of those principles which have come down to us from remote times, before surgery was finally established on its present basis, and to show you the ancient root and stem from which the branches of to-day have grown. We may thus together help to pay off a debt which is insufficiently acknowledged, and perhaps be led to exclaim, verily "there is no new thing under the sun." In our medical library there are some very old books containing plates which will serve to illustrate my subject.

For the earliest records of surgery we must go to the wonderland of Egypt. *Athotis* (Thosortes), the son of Menes, the first King of Egypt, probably reigned about B.C. 3500 or earlier, and his medical knowledge was sufficiently extensive for him to receive the surname of "Imhotep," *i.e.* "physician." His temple was at Memphis, and his position among the Egyptians was much the same as that of Æsculapius among the Greeks.

About this time the physicians were guided, under penalty of punishment, by the directions contained in the so-called "Hermetic Books," the originals of which have not been discovered; but some of the contents are probably preserved to us in some ancient papyri, of which one of the best known is the papyrus of Ebers. This interesting manuscript was found between the bones of a mummy at Thebes, and was written about B.C. 1552, when Moses was twenty-one years of age, and therefore before the exodus of the Israelites from Egypt. It is written in hieratic characters—a cursive form of the hieroglyphic method of writing—and was probably compiled at Heliopolis, where there was a school of practical medicine, as there was also at Thebes, Memphis and other places. The work gives directions for the cure of diseases of the eyes, ears and tongue, but treats mainly of medical subjects.

The physicians of the Pharaohs were, however, familiar with such operations as amputations and circumcision; and the great antiquity of the latter is shown by the fact that the operator generally used a flint knife in accordance with ancient custom. As there are no representations of Egyptian circumcision earlier than the time of Rameses II (B.C. 1310–1243), it is probable that the circumcision of Abraham about the year B.C. 1897, as narrated in the Scriptures, is the earliest record of a definite surgical operation.

The ancient Egyptians did little to advance either surgery or anatomy, and, putting aside other early civilisations, we must now turn to ancient Greece, with which the history of surgery in the west is more particularly connected.

The Grecians regarded medical practice as a branch of that philosophy and art for which they are so justly famous. The cult of *Asklepios*—or, to take the more usual Latin equivalent, *Æsculapius*—existed before the destruction of Troy, which occurred about B.C. 1200. It is not unreasonable to infer that Æsculapius and his sons Machaon and Podalirius were real personages renowned for their medical skill; and a stone tablet found in the Hieron Valley, near Epidaurus, where Æsculapius is said to have been born, seems to show a group of people asking the advice of Æsculapius and his family.

According to Homer, Æsculapius and his sons were engaged in surgical work, acting like the army surgeons of modern times. Podalirius and Machaon were at the siege of Troy, and were employed in such operations as extracting barbed arrows by incision, and stopping hemorrhage from wounds.

Machaon is credited with curing Philoctetes of a foul ulcer by cutting out the wound, washing it with wine, and applying healing herbs. This use of wine by the ancients was very common. Doubtless the good it effected was due to the antiseptic properties of the alcohol contained in it, and at the present time a solution of alcohol is one of our valued antiseptics.

In Homer no mention is made of any divine origin of Æsculapius—he is simply the "blameless physician"; but after his death wonderful birth-stories arose, as in the case of Alexander and other great men of ancient times.

These legends ascribe the parentage of Æsculapius to Apollo and Coronis, and he is said to have been born by Cæsarean section just before his mother was burnt in the flames to which the jealous Apollo had committed her.

Many temples were erected in honour of Æsculapius after his deification, those at Epidauros, Cnidos and Cos being amongst the most important. At these temples the officiating priest—who might or might not be a physician—brought the sick person before an image of the god, and the necessary sacrifices were made accompanied by prayers and hymns and other ritual.

Snakes and dogs—both of which were sacred to Æsculapius—are said to have been trained to lick ulcers or other diseased conditions with their tongues. These snakes, which were quite harmless, were peculiar to Epidauros, where they abounded. Sometimes the god was said to appear in the form of a serpent, and the staff of Æsculapius is represented with a snake, and remains as the symbol of the medical profession to this day.

Guilds of lay physicians, known as Asclepiadæ, were formed, and these, rather than the Æsculapian priests, became the direct founders of Grecian medicine.

The Asclepiadæ were great travellers, and by this means gained most of their education from the noted men whom they met, and their medical knowledge was bequeathed from father to son by oral tradition until the time of Hippocrates.

There were, however, schools for medical instruction and practice founded by the Asclepiadæ, and it was at the conclusion of their course of study that the pupils were required to subscribe to the declaration which is commonly known as the Hippocratic oath, but which is of older origin. The two most celebrated of these schools were at Cnidos, in Asia Minor, and Cos, an island in the Ægean Sea.

Pl. 1.
*Hippocrates the Great.*

In these days the physicians practised both medicine and surgery, though there were irregular practitioners who specialised as lithotomists, oculists or dentists. Medical knowledge was regarded as a secret of the guilds, and many of the guilds were dominated by priestly influence.

Much of this was altered by *Hippocrates the Second*, or "Great," whose surgical knowledge and practice were based on observation and experience. He taught publicly, and founded a system of medicine free from sacerdotalism (Pl. 1).

Born in the island of Cos, in B.C. 460, Hippocrates the Second was the son of the Asclepiad Heraclides, and claimed to be seventeenth in descent from Æsculapius. He therefore lived in one of the most remarkable periods of intellectual development that the world has known. Pericles the famous Athenian statesman, Socrates the philosopher, Herodotus the historian, and Phidias the greatest sculptor of ancient Greece, with many other distinguished men, were his contemporaries ; and doubtless the spirit of philosophic inquiry which was then so general influenced Hippocrates in the formation of his rational ideas on medicine and surgery. Hippocrates practised in Cos, Delos, Athens and other places, and finally retired to Larissa, in Thessaly, where he died about B.C. 370.

He was the first to commit his medical knowledge to writing, and his work has come down to us as a record of the surgery which was then practised with so high a degree of operative skill.

In his treatise on injuries of the head, Hippocrates divides injuries of the cranial bones into (1) simple fractures or fissures, (2) contusions, (3) depressed fractures, (4) fractures of the outer table only, and (5) the counter-fissure, or, as we call it, fracture by "contre-coup."

Trepanning was practised for simple, but not depressed, fractures and for the removal of accumulations of pus and blood ; and he warns us against the use of the trephine over the sutures. The modiolus or ancient trephine, the terebra or trepan called abaptiston, the forceps, raspatory, lenticular and mallet were all much the same as those in use at the present day. He knew, too, that injury of one cerebral hemisphere resulted in spasm or paralysis of the muscles on the opposite side of the body. The thorax was opened for empyema and hydrothorax, paracentesis abdominis was practised, and liver abscess and suppuration in the kidney were treated by incision.

The training for the public games which took place in Olympia doubtless made him familiar with fractures and dislocations, and his treatises on these subjects are remarkably complete and accurate.

Dislocations of almost all the articulations are described, and his account of those of the hip-joint shows a power of observation that most of us would be glad to possess. He minutely records the symptoms of dislocation of the hip-joint, whether congenital, traumatic, or resulting from disease; and not only does he describe the four ordinary dislocations, namely upwards, downwards, forwards and backwards, but he also gives us an excellent account of the appearances of the limb when the dislocation has been left unreduced. He clearly understands the method of reduction by manipulation, for he tells us that in some cases "the reduction is effected by bending the limb at the joint and making rotation." He describes the method of reduction by extension and counter-extension, and in other cases employed levers attached to the scamnum Hippocratis upon which the patient was placed, or used extension over the rung of a ladder.

Pl. 2.

*Reduction of dislocation of the shoulder by means of the ambe.*

Downward dislocation of the shoulder is the only form with which Hippocrates was familiar, and he tells us that he had "never seen it take place upwards or outwards." We are told how to reduce shoulder dislocations by different methods, such as the use of the ambe (Pl. 2) or the heel in the axilla. "The patient must lie on the ground upon his back, while the person who is to effect the reduction is seated on the ground upon the side of the dislocation; then the operator, seizing with his hand the affected arm, is to pull it, while with his heel in the armpit he pushed in the contrary direction, the right heel being placed in the right armpit, and the left heel in the left armpit."

In the treatment of fractures waxed bandages—the forerunner of our plaster-of-Paris bandages—were used after reduction by extension and counter-extension; but in cases which did not admit of treatment by the various methods of bandaging which he describes, an ingenious splint was used to keep the broken limb extended. This splint consisted of upper and lower rings of leather, through which the affected limb was placed, and the rings were kept apart by rods of cornel or dog-wood of suitable length, so that when adjusted they produced "a proper and equable extension in a straight line, without giving any pain to the wound." In other cases the fractured limb was placed in a canal or gutter of wood. The reduction of compound fractures, the sawing off of protruding ends of bone, the occurrence of exfoliation, and the dangers of these conditions, are fully discussed.

Pl. 3.

*Reduction of fracture-dislocation of the spine by means of a lever attached to the scamnum Hippocratis.*

His knowledge of club-foot was remarkable; but his words were forgotten, and it is only in recent times that any advance was made on his method of treating this deformity. Hippocrates knew the exact nature of this affection, and tells us that in the treatment of congenital cases we should begin "at as early a period as possible." His plan consisted in restoring the position of the bones as far as possible by the hands, and securing the parts by means of soft bandages and a mixture of wax and resin. He also used a form of leaden shoe, "having the same shape as the Chian slippers had," and speaks of the necessity of wearing some such support after the foot has been restored to its proper shape.

By a rough method of succussion—after fastening the patient to a ladder, which was allowed to fall from a height—the spine was straightened in cases of fracture-dislocation ; but Hippocrates tells us that he preferred to remedy the deformity by means of a lever (Pl. 3), or by pressure with the hands, in much the same way as Calot suggested for spinal caries a few years ago (Pl. 4).

Venesection and cupping were performed, the rectum was explored by a speculum, and fistula and hæmorrhoids were cured by operation. He gives us minute directions for different forms of bandage, and deals with such subjects as the relative position of the patient and operator, the light, instruments, and so forth.

Pl. 4.

*Reduction of fracture-dislocation of the spine by manipulation.*

Considering the anatomical knowledge of Hippocrates was practically limited to the bones, and a superficial acquaintance with the viscera, his surgical achievements are all the more wonderful. Thoroughness and boldness were the characteristics of this Father of Surgery, who transmitted to the world an imperishable name and work. He laid the first foundations upon which others built, and we can fully endorse the action of the inhabitants of Argos, who voted him a statue of gold.

After the death of Hippocrates there is little to record of surgical importance in Greece ; but *Praxagoras* of Cos (about B.C. 335), the teacher of Herophilus, and the last of the Asclepiadæ, used taxis for strangulated hernia, and recommended, if he did not actually perform, abdominal section, removal of the obstruction and suture of the intestine, where purgatives, enemata and massage of the abdomen had failed to relieve obstruction of the bowels.

The cradle of surgery now changes from Greece to Egypt.

Greece itself had come under the dominion of Philip of Macedon, who was succeeded by his son, Alexander the Great; and the latter, having defeated the Persians in Asia Minor, found no difficulty in subjugating Egypt. He founded Alexandria in B.C. 332, and under the Ptolemies this ancient capital soon became the most splendid and wealthy city of the known world. The famous School of Alexandria was established with Aristotle, (Alexander the Great's former teacher) as the Master about B.C. 300. It maintained some of its importance till the time of Hypatia, who was cruelly slaughtered in A.D. 415, and continued to exist until Alexandria fell into the hands of the Arabs in A.D. 640.

Pl. 5.

*Scarification of legs for dropsy.*

*Herophilus* (about B.C. 335–280), the founder of human anatomy, was one of the earliest members of the School of Alexandria, and his contemporary, *Erasistratus*, opened the abdomen for the purpose of directly applying remedies to the surface of the liver and spleen, and invented a catheter shaped like the letter "S." But it is probable that some form of catheter was also employed by the immediate followers of Hippocrates.

The development of anatomy had a beneficial effect upon the progress of surgery at the Alexandrian School, but probably much valuable information on the subject was lost when a large part of the library of 700,000 volumes was destroyed by fire during the siege of Alexandria by Julius Cæsar in B.C. 48.

With the tragic death of Cleopatra in B.C. 30 the dynasty of the Ptolemies came to an end, and Egypt became a Roman province. After this event Alexandria declined in importance, and as Rome became the centre of civilisation, we must now trace the development of surgery in the Roman capital; but we must remember that it is still essentially Greek in substance, even when maturing under Roman influence.

Pl. 6.
*Celsus.*

For many hundreds of years the practice of medicine was regarded as dishonourable by Romans of the upper class; but the prejudice against practitioners of the healing art was much diminished by the success of *Asclepiades* (B.C. 128–56), a native of Bithynia, who after studying at Alexandria settled in Rome, and became the friend of Cicero and other distinguished men of his time. His name is memorable as that of the first surgeon who practised tracheotomy. He also observed the occurrence of spontaneous dislocations in disease, and recommended scarification of the ankles in dropsy (Pl. 5).

The most famous Roman writer on medical subjects is *Celsus* (Pl. 6), and his treatise is the earliest that has come down to us in the Latin language. He lived (about B.C. 30 to A.D. 50) in the golden age of Augustus and Tiberius, and though some doubt has arisen as to whether he actually practised medicine or not, his knowledge of the art seems to have justly earned for him the title of the Latin Hippocrates. From him we learn of the advances made in surgery by the Alexandrian School since the time of Hippocrates.

He tells us that " a surgeon ought to be young, or at all events not much advanced in years ; possessed of a hand that is firm, steady, uniformly devoid of tremor ; he should be able to use both hands with equal dexterity ; his sight should be penetrating and clear, with a mind undaunted ; he should retain so much compassion as may lead him to wish for his patient's recovery, without being so far moved by his shrieks as to induce him to make unnecessary haste, or to cut less than is absolutely expedient . . ."

He recommends that fistulæ should be laid open and their callous portions excised, and accurately describes the needling of cataracts ; he deals with plastic surgery of the ears, lips and nose, and uses sutures to approximate the pared edges of a hare-lip. He warns us against fracture of the alveolar margin in extracting teeth, and wound of the ranine vessels in dividing the frenum linguæ: in the treatment of bronchocele he enucleated cysts after incision.

Wounds of the intestine are dealt with, and though he regarded a wound of the small gut as irremediable, he advocated suture of the colon, " for sometimes reunion is effected." The abdominal parietes were closed by suturing the peritoneum separately from the other layers, and a sponge moistened with vinegar was applied to the incision beneath a bandage. Reducible herniæ were treated with pads and bandages, and open operations were performed for strangulated hernia, hydrocele and varicocele.

After an excellent description of the manner of passing copper catheters, and the removal by incision of an impacted urethral calculus, he gives us his method of cutting for stone in the bladder.

Owing to want of success, the operation of lithotomy was confined in early days to irregular practitioners, who were regarded as disreputable, and surgeons were forbidden to cut for stone by the Hippocratic oath. Celsus placed the operation on a firm basis, and his method of "cutting on the gripe," as it was also called, was followed for nearly sixteen hundred years. After fixing the stone by passing a finger into the rectum, the neck of the bladder was opened by means of an incision in the perineum. For extraction of the calculus the finger, or an instrument known as the crotchet, was used. He further states that "should the stone appear too large to admit of extraction without rupturing the neck of the bladder, it must be split."

Pl. 7.
*Amputation of leg.*

The credit of this suggestion he gives to Ammonius of Alexandria, surnamed "Lithotomos," who lived about B.C. 230.

In amputation for gangrene there is also a great advance over the Hippocratic method, which only allowed the dead portions to be cut away after a line of demarcation had formed. Celsus, however, tells us to make a circular cut into the "sound part, rather than to leave any of that which is diseased." The bone should be sawn close up to the healthy flesh, and the stump covered as far as possible with the divided integuments, which should be long enough for this purpose (Pl. 7).

One more example may be given of the advance in surgery in the time of Celsus, who distinctly prefers the use of the ligature to that of the cautery iron in the treatment of hemorrhage. After speaking of the use of cold water, vinegar and other means of arresting hemorrhage, he says: "But should these means fail also, the bleeding vessels should be taken up, and ligatures having been applied above and below the wounded part, the vessels are to be divided in the interspace; that thus they may retract, while their orifices yet remain closed," a practice which in itself would have been sufficient to have immortalised this wonderful surgeon of the early Christian Era.

At the end of the first century *anno Domini*, *Heliodorus* gave us the first account of the terminal ligature and the torsion of arteries. He says: "We ligature the large vessels; but as for the smaller ones, we catch them with hooks, and twist them many times, thus closing their mouths." He also described the isolation and removal of the sac in hernia, and is said to have been the first to perform internal urethrotomy for stricture.

About the same time *Archigenes* (A.D. 48–117) placed amputation of the limbs on a better basis by ligaturing the main vessels as a preliminary measure when it was impracticable to bandage the limb and fasten a tight cord above the area of operation, after the manner of the rubber bandage and tourniquet of Esmarch in modern times.

*Claudius Galen* (A.D. 131–201) was born at Pergamus, and like many of his predecessors studied at Alexandria before practising in Rome (Pl. 8). His knowledge covered a wide field in science, rhetoric and philosophy, and being an indefatigable worker, he collected all that was known of medicine before his time. His works exceed three hundred in number, and his influence in medicine, like that of Aristotle in philosophy, continued down to the fifteenth century.

He did not, however, add much to surgical knowledge, in which he mainly followed the teaching of Hippocrates. His chief operation seems to have been that of bleeding, but he was adept in bandaging, and introduced those forms known as the accipiter, sling, spica, etc. He appears to have had a fondness for mechanical appliances, and in the reduction of dislocations and fractures he used the unwieldy machinery of bygone times.

His reputation as an anatomist is of a higher order, though his observations were made on the lower animals, except in the case of the bones, which he studied on human skeletons at Alexandria. He seems to have dissected anything from an elephant to a mouse, but much of his excellent work on the nervous system was based on vivisection, for the ancients objected less to this method of investigation than to interference with the dead, and human anatomy was almost entirely neglected except in Alexandria.

Pl. 8.
*Galen.*

*Antyllus* was a distinguished Italian surgeon who flourished about the year A.D. 200. He was the first to give an accurate account of aneurysm, and invented the operation for its cure which goes by his name, and is practised to this day—ligature above and below the aneurysm, with incision of the sac and evacuation of the clot. He also performed subcutaneous section of the ligaments in stiff joints and the extraction of cataracts.

From this time surgery began to decline in Rome, for the luxury, extravagance and vice of the Romans—in spite of the rule of Constantine the Great (A.D. 306–37) who had become a Christian—were incompatible with the encouragement of philosophy, science or art.

The importance of Rome as a centre of learning was still further diminished by Constantine's removal of the seat of empire to Byzantium, which was dedicated to him under the new name of Constantinople in A.D. 330.

The only writer of any importance in this period was *Oribasius* (A.D. 326–403), of Pergamus, who wrote in Greek, and was the last of the great pagan medical authors. He was physician to Julian the Apostate, and at his request Oribasius made an epitome of the works of Galen. He was an assiduous collector of the medical knowledge of previous ages, and it is to his writings that we are indebted for the account of Antyllus, Heliodorus and others that have been referred to.

With the overthrow of the Western Empire by Odoacer, King of the Heruli, in A.D. 476, we enter upon the mediæval period, and for some hundreds of years the progress of surgery was delayed. It will suffice, therefore, if I only briefly refer to some of the more important advances made by those who helped to illumine these dark ages of surgery.

The spread of Christianity had placed great power in the hands of its converts, but the ceaseless theological discussions accompanying its early growth left little time for the advancement of other knowledge. Medicine passed largely into the hands of the priests, and surgery was neglected.

Educated Greeks of Constantinople, who had studied at Alexandria, fortunately continued to keep alive the knowledge of the ancients by writing compilations of their works, and to these they added any further information which had been acquired up to their own time.

One of the earliest of these eastern physicians was *Aëtius*, of Amida (now Diarbekir) in Mesopotamia (A.D. 502–75), who was evidently a Christian, for if a bone which had stuck in a patient's throat could not be extracted by means of a piece of meat, attached to a string, which the patient had swallowed, he directed the physician to grasp the patient by the throat and cry in a loud voice: "As Jesus Christ drew Lazarus from the grave, and Jonah out of the whale, thus Blasius, the martyr and servant of God, commands 'Bone come up or go down.'"

His voluminous writings, however, contain a good deal of valuable information which is not found in the works of Galen and Celsus, and he was evidently familiar with the advantages of treating an aneurysm by ligaturing the artery at some distance above it. Thus, when speaking of aneurysm at the bend of the elbow, he tells us to proceed as follows: "In the first place, having marked the course of the artery from the armpit to the forearm, we are to make a simple incision, three or four fingers' breadth below the armpit, where the artery is most superficial; and having laid it bare and separated it from the surrounding parts, to seize the artery with a blunt hook and bind it with two ligatures, after which it is to be divided between them, and the wound filled with fine frankincense, and a suitable pledget of lint bound over it." Like other ancient writers, he regarded carotid, axillary and inguinal aneurysms as incurable.

He distinguished several varieties of hernia, made use of the uterine, sound and sponge tents, and gave clear directions on the surgical treatment of cancer of the breast, using the cautery not only to stop the hemorrhage but also to destroy every particle of diseased tissue.

The greatest of these Byzantine surgeons was *Paul of Ægina* (A.D. 625–90), who was deservedly famous in both surgery and gynæcology. So great was the esteem of the Arabians, that his writings were translated by them, and became one of their chief medical text-books.

His sixth book, which treats of surgery, is considered by many to be the best account of surgical knowledge previous to the revival of learning at the end of the Middle Ages.

Paulus Ægineta, as he is generally called, practised venesection, arteriotomy and cupping, and for the last operation he mentions cupping instruments of glass, copper and horn. In lithotomy he does not confine the operation to children as Celsus did, but allows its propriety at any age. His incision was distinctly lateral, and in this he again differs from the method of Celsus. He says: "Between the anus and the testicles, not however in the middle of the perineum, but on one side, towards the left buttock, we make an oblique incision, cutting down direct upon the stone where it protrudes, so that the external incision may be wider, but the internal not larger than just to allow the stone to fall through it."

His method of the ligature of varicose veins is the same as the operation of to-day, even to the marking of the veins distended by directing the patient to walk about before operation; and Paulus also mentions that others used to tear the veins out by force, a statement particularly interesting to those of us who live in Bristol and still practise avulsion of varicose veins.

His account of the extraction of weapons is fuller than that of previous writers, and contains much of interest. He tells us that "when the membranes of the brain or cerebrum itself is wounded, we extract the weapon by trepanning the skull. . . . If the weapon is lodged in the chest, and does not come out readily, it is to be extracted by means of a moderate incision in the intercostal space, or by cutting out a rib with the assistance of the instrument called meningo-phylax. In like manner, when the stomach, bladder and other deep parts are wounded, if the weapon comes out readily it is to be extracted without more ado; but if not, we must enlarge the wound. . . . In wounds of the abdomen, gastroraphé as formerly described may be had recourse to if necessary. But if the weapon has lodged in any of the larger vessels, such as the internal jugulars or carotids, and the large arteries in the armpits or groins, and if the extraction threaten a great hemorrhage, they are first to be secured with ligatures on both sides, and then the extraction is to be made."

In badly united fractures he recommended re-fracture even with the use of the chisel, treated hydrocephalus by puncture, and mentioned extirpation of the uterus and the dilatation of strictures of the rectum by means of bougies.

Paulus Ægineta was one of the last Greek medical writers, and the last important student of the famous School of Alexandria.

A new era now begins in the history of surgery, for while the advance of learning was checked in Europe, the newly-arisen Mohammedan powers in Asia became the keepers and transmitters of the scientific knowledge of the Greeks.

Mohammed died in A.D. 632, but the spread of his religious opinions at the point of the sword led to the establishment of an empire greater than that of Alexander. Jerusalem fell into the hands of the Saracens in A.D. 637, and the famous city of Alexandria shared the same fate in A.D. 640, after a siege of fourteen months, when its celebrated library was again largely destroyed.

Before this period the Arabians had become familiar with the literature of ancient Greece, not only by their commercial relations with Alexandria, but also by the formation of Jewish schools in western Asia—most of which owed their foundation to Alexandrian influence—and the Christian schools in Mesopotamia, as the result of the banishment of the Nestorians in A.D. 439, because the Patriarch of Constantinople, from whom they took their name, had inculcated the doctrine that Mary should be called "Mother of Christ," and not "Mother of God."

Jews and Nestorians translated Greek authors into Syriac, with which many of the Arabians and Persians were familiar.

With the extension of Saracenic influence, after the fall of Alexandria, important manuscripts were carefully collected and translated into Arabic, and schools were founded after the model of that at Alexandria. Bagdad became celebrated as the seat of learning, but schools and hospitals were also established in Persia, Egypt, Spain, and other countries.

The Greek authors previously mentioned were the chief favourites, and so the surgery of the Arabians was essentially that of the Greeks; but if the Arabians did little to advance our art, we are at least indebted to them for the care which they took in preserving the knowledge of previous ages while the nations of Europe looked on with indifference.

The Arabians faithfully discharged their trust until the destruction of Bagdad by the Mongols in 1258, but their influence continued until the seventeenth century.

*Rhazes* (A.D. 850–923), one of the earliest of the Arabic writers, was born in Persia, and became a practitioner and teacher at Bagdad. He is the first to describe spina bifida, and advised that catheters should have lateral holes to render them less easily obstructed by clots. He practised puncture of the bladder, and removed the entire tibia and humerus in cases of necrosis.

*Haly Abbas* (A.D. 930–94), another Persian who lived in Bagdad and wrote in Arabic, recommended the excision of strumous glands and the rupture of ganglionic cysts by striking them with a hard body.

*Avicenna*, "the Prince of Physicians" (A.D. 980–1037), was held in greater esteem than his predecessors, and his *Canon* was the chief text-book for European medicine for more than five hundred years. He is, however, more famous as a philosopher than a surgeon, but appears to have been the first to use obstetric forceps for the extraction of the child in cases of difficult labour, and thus preceded the "introduction" of midwifery forceps by the Chamberlens by some six hundred years.

The fame of these writers of the eastern caliphate was rivalled by the Spanish-Arabian surgeons in the western caliphate, particularly those of Cordova.

Pl. 9.
*Drill for breaking an impacted calculus.*

*Albucasis* (A.D. 1060–1122 ?), whose *Compendium* is largely founded on the work of Paulus Ægineta, gives the earliest drawings of surgical instruments; but, like most of the Arabians, he was an over-cautious operator, and relied greatly upon the actual cautery. He sutured the intestines with threads scraped from the intestinal coats, and used a silver catheter in place of the copper instrument previously in vogue.

*Avenzohar* (A.D. 1113–62), the teacher of the celebrated Averroës, was born in Seville, and was honoured by the title of "The Wise and the Illustrious." He mentions the use of a silver or tin œsophageal tube for the purpose of artificial feeding, and recommended the use of the trephine in depressed fractures of the skull. He was also the first to attempt to diminish the size of an impacted urethral calculus by means of a sort of drill passed into the urethra, the prototype of the modern lithotrite (Pl. 9).

With the capture of Cordova in 1236 by Ferdinand III of Castile, and that of Bagdad in 1258 by the Mongols, the further development of surgery among the Arabians practically ceased, though the influence of their accumulated knowledge of the subject continued till the seventeenth century.

Meanwhile the progress of surgery in Europe had greatly suffered with the extension of Christianity. In the earlier part of the Middle Ages the practice of surgery was neglected by the priests, who had become the medical practitioners of these times; but a knowledge of the Greek, Roman and Arabic writers was fostered by the teachers and students of the celebrated School of Salerno, which was probably founded in the ninth century, or earlier, and was practically free from clerical influence.

Ultimately the healing art was divorced from the priesthood, and in the thirteenth century most of the famous universities of Europe—such as those at Paris, Montpellier, Padua and Oxford—were founded.

This led to a revival of human anatomy under *Mondino*, of Bologna (1276–1326), and produced a number of men of independent thought, who became acquainted with the surgery of the ancients largely through the writings of the Arabians.

The teaching and works of such men as *William of Salicet* (1201–80), professor at Bologna and Verona; *Arnald of Villeneuve* (1235–1313), *Bruno of Calabria* (about 1252), and *Henry of Mondeville* (died about 1315), a teacher at Montpellier and Paris, did much to restore the science and art of surgery in accordance with principles which had been too long neglected. *John Ardern*, a practitioner of Newark, in Nottinghamshire, and sergeant-surgeon to Edward III in 1346, deserves special mention, as he was the first surgical author of importance in England.

But the greatest of all the surgeons of this Arabic revival was *Guy de Chauliac*, who was born about 1300. His *Chirurgia Magna* passes in review the writings of the Greek and Arabian authors, of which his work is confessedly a compilation (Pl. 10). There is, however, much original matter in his book, which was published in 1363, and which continued to be the chief text-book in France for many years after his death.

He knew the beneficial effect of completely dividing half-severed vessels which continued to bleed, used a sort of cradle for the suspension of fractured limbs, and treated fracture of the thigh by means of a weight and pulley, in addition to the long splint. He had an extensive knowledge of diseases of the eye, and recommended spectacles in cases of weak sight. Spectacles were, however, introduced by Roger Bacon (1214–92), but probably they were invented by the Arabians, with whose science he was familiar.

Pl. 10.
*A lecture in anatomy in the fourteenth century.*

Guy de Chauliac gave clear indications for trephining, removed pharyngeal polypi, united wounds in order to get primary healing, treated ulcers by compressive bandages, and even recommended that a rope should be placed over the patient's bed to help him in raising himself.

The Arabo-scholastic revival of which I have been speaking was followed by that renewed interest in classical learning in which our medical confrère *Linacre* (1461–1524) played such an important part; and the Middle Ages may be said to close with the invention of printing (Coster, 1440; Caxton, 1476), and the discovery of America (1497) by John Cabot, who sailed from this port of Bristol.

With the modern era I am not concerned to-night. My endeavour has been to show how extensively the surgery of to-day is dependent upon the labours of ancient and mediæval practitioners of our art. The time at my disposal has prevented me from mentioning many names worthy of our remembrance, and I am very conscious of the scant justice I have done to the work of those to whom reference has been made; but I hope that, at least, enough has been said to save us from egotism, ingratitude and forgetfulness.

### REFERENCES.

Most of the books consulted are appended, and from those mentioned earlier in the list I have borrowed freely.

BAAS, JOH. HERMANN. *Outlines of the History of Medicine and the Medical Profession.* Translated, and in conjunction with the author, revised and enlarged by H. E. Handerson. 1889.

FISHER, G. J. "A History of Surgery." *Ashhurst's International Encyclopædia of Surgery.* 1886. Vol. VI.

HIPPOCRATES (COUS.), Genuine Works. Translated from the Greek by Francis Adams. 2 vols. (Syd, Soc.) 1849.

HIPPOCRATES (COUS.). *The Aphorisms of Hippocrates, and the Sentences of Celsus. To which are added Aphorisms upon several distempers not well distinguished by the Ancients* By Sir Conrad Sprengel. 1735.

PAULUS ÆGINETA, The seven books of. Translated from the Greek by F. Adams. (Syd. Soc.) 3 vols. 1844–47.

CATON, RICHARD. *The Temples and Ritual of Asklepios at Epidauros and Athens.* 1900.

WITHINGTON, EDWARD THEODORE. *Medical History from the Earliest Times: A Popular History of the Healing Art.* 1894.

MACFIE, RONALD CAMPBELL. *The Romance of Medicine.* 1907.

CATON, RICHARD. *I-em-hotep, and Ancient Egyptian Medicine.* Harveian Oration. 1904.

REMONDINO, P. C. *History of Circumcision from the Earliest Times to the Present Moral and Physical Reasons for its Performance, with a History of Eunuchism, Hermaphrodism, etc., and of the Different Operations Practiced upon the Prepuce.* 1891.

CELSUS, AUL. CORN. A Translation of the Eight Books of Celsus on Medicine. 3rd Edition. Carefully revised and improved by G. F. Collier. 1838.

RIOLLAY, F. *Doctrines and Practice of Hippocrates in Surgery and Physic.* 1783.

DENNIS, F. S. *System of Surgery.* 1895.

SOUTH, J. F. *Memorials of the Craft of Surgery in England.* Edited by D'Arcy Power. 1886.

POWER, D'ARCY. "How Surgery became a Profession in London." Reprinted from *Medical Magazine,* 1899.

PARE, AMBROISE. *The Works of that famous chirurgeon, Ambrose Parey. Translated out of the Latin, and compared with the French by Th. Johnson.* 1678.

FABRICIUS AB AQUAPENDENTE HIERONYMUS. *Opera chirurgica.* 1723.

HILDANUS, GUILHELMUS FABRICIUS. *Opera observationum et curationum medico-chiurgicarum quæ extant omnia (additus Marsus Aurelius Severinus de efficicaci medicina).* 1646.
DIONIS (PIERRE). *Cours d'Opérations de Chirurgie, demontrées au Jardin royal.* 1708.
PARK, ROSWELL. *An Epitome of the History of Medicine.* 1899.
SCULTETIUS, JOHANNES. *Armamentarium Chirurgicum. Studio Johannis Tilingii.* (With Appendix.) 1693.
ANDERSON, W. Introductory Address, "History of European Surgery." *St. Thomas's Hospital Reports.* 1890. xviii, p. 23.
GALEN, C. [*Opera Omnia.*] 4 vols. [Venice.] 1562.
FALLOPIUS, G. *Expositio in librum Galeni de ossibus huic accesserunt observationes anatomicæ eiusdem authoris.* 4to. Venice. 1570.
JAMES, R. *Dictionnaire Universel de Médecine de Chirurgie, etc.* Trad. de l'Anglois de R. James par Diderot, eidous et Toussaint. 6 vols. Folio. 1746–48.
HILDANUS, G. FABRICIUS. *Opera observationum et curationum quæ extant omnia.* 1646.
HIPPOCRATES (COUS) and GALENUS (CLAUDIUS). *Opera: Renatus Charterius emindavit et Graece et Latine primus edidit.* 13 vols. in 9. Fol. 1679.
GUY DE CHAULIAC. *La Grande Chirurgie.* composée En l'an, 1363. Paris, 1890.
HALLER, ALBERTUS. *Disputationes Chiurgicæ Selectæ.* 5 vols. Amsterdam. 1755-56.
ALPINUS, PROSPERUS. *Præsagienda vita et morte ægrotantium libri septem.* Lugd. Bat. 1733.
MACCALLUM, W. G. "The School of Montpellier." *Johns Hopkins Hosp. Bull.* 1908. xix, p. 296.
SOZINSKEY, THOMAS S. *Medical Symbolism in connection with Historical Studies in the Arts of Healing and Hygiene.* Philad. 1891.
RICHARDS, JOHN MORGAN. *A Chronology of Medicine.* Lond. 1880.
ALPINUS, PROSPERUS. *Medicina Ægyptiorum.* Editio Nova. Ludg. Bat. 1719.

## Figures and Tables

**Pl. 1. f1:**
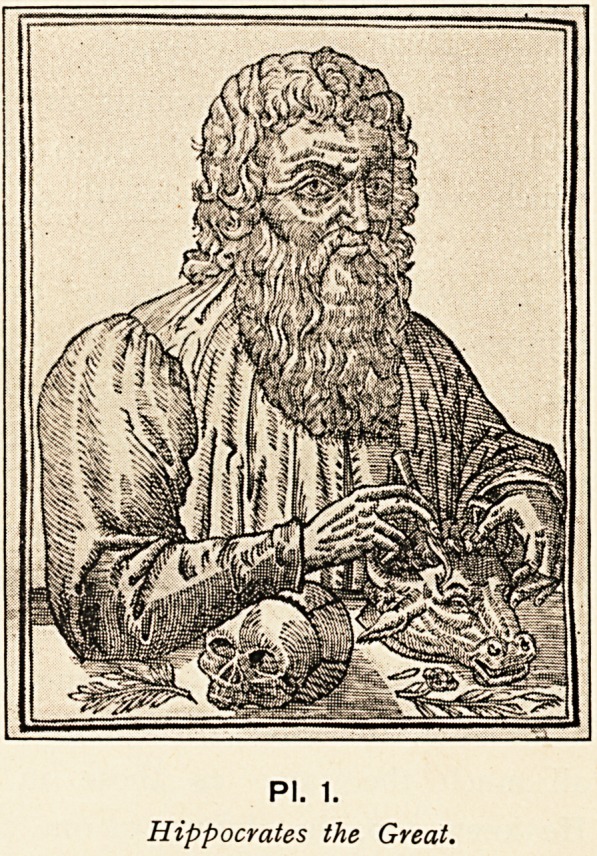


**Pl. 2. f2:**
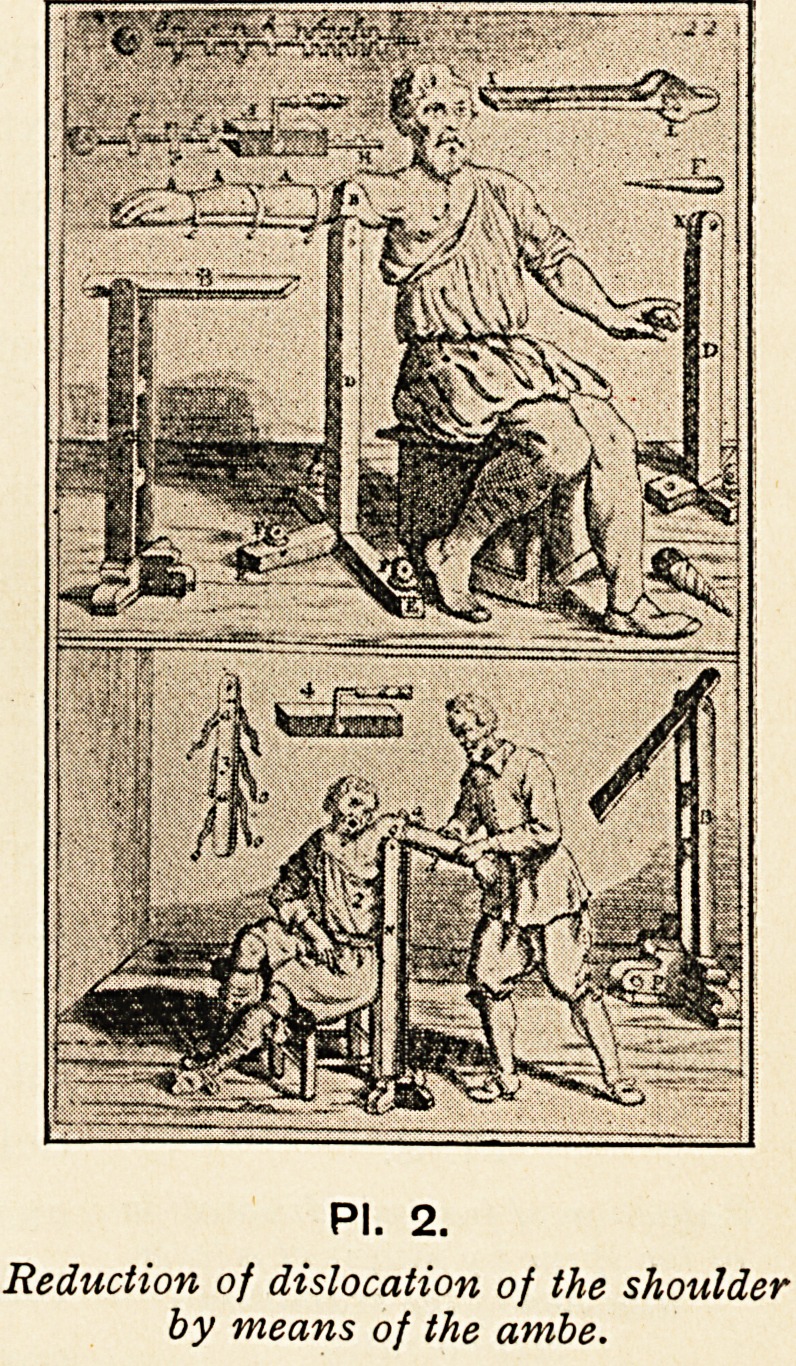


**Pl. 3. f3:**
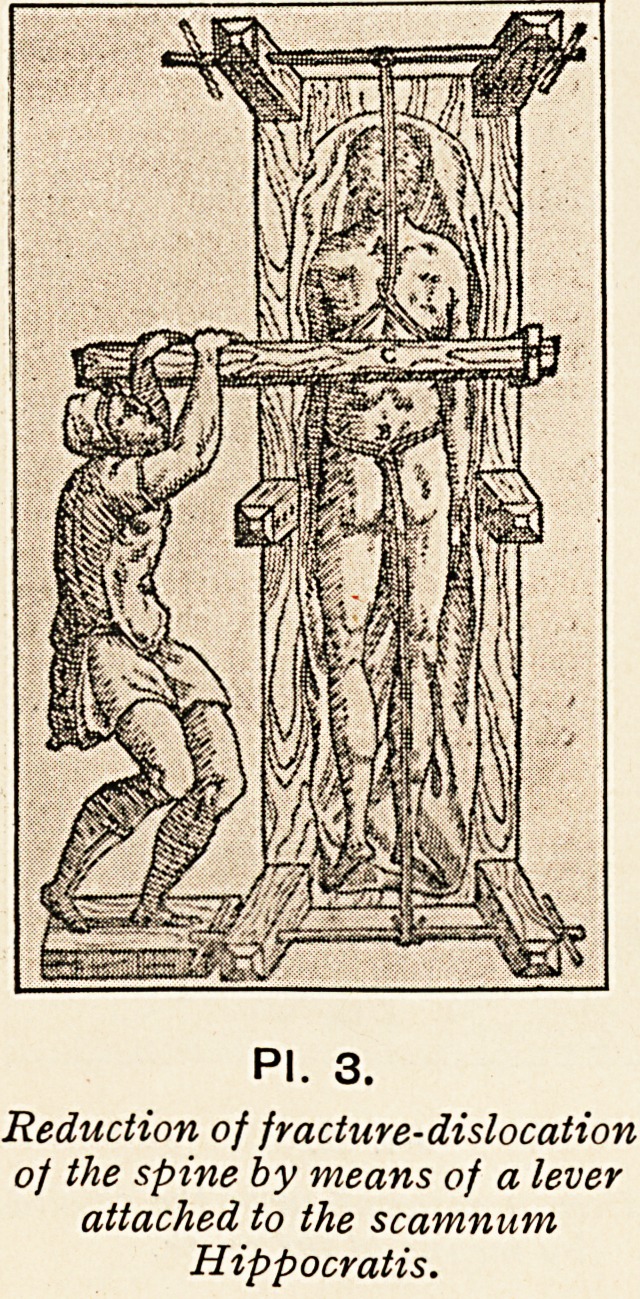


**Pl. 4. f4:**
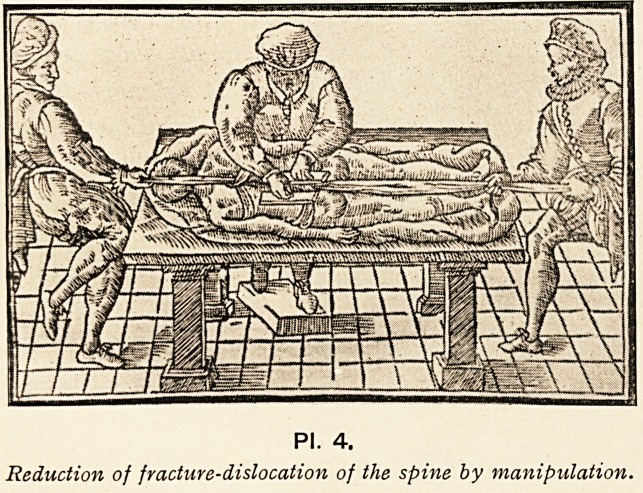


**Pl. 5. f5:**
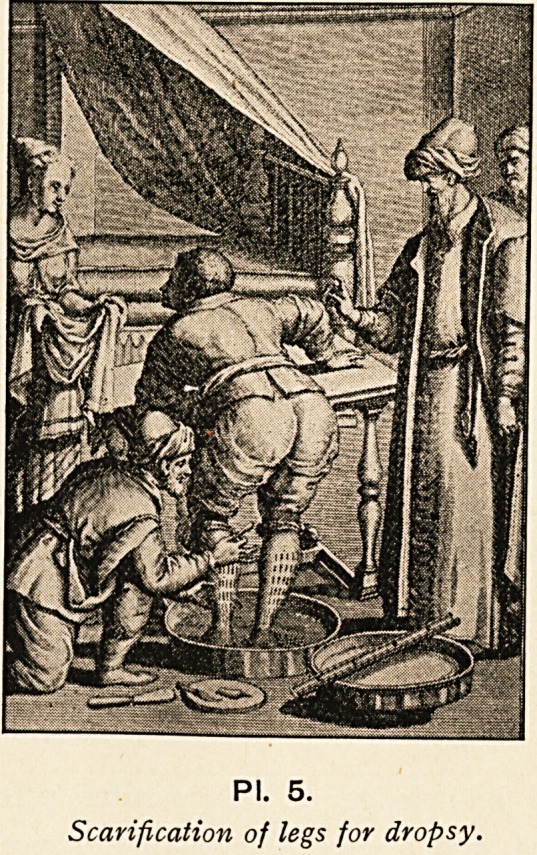


**Pl. 6. f6:**
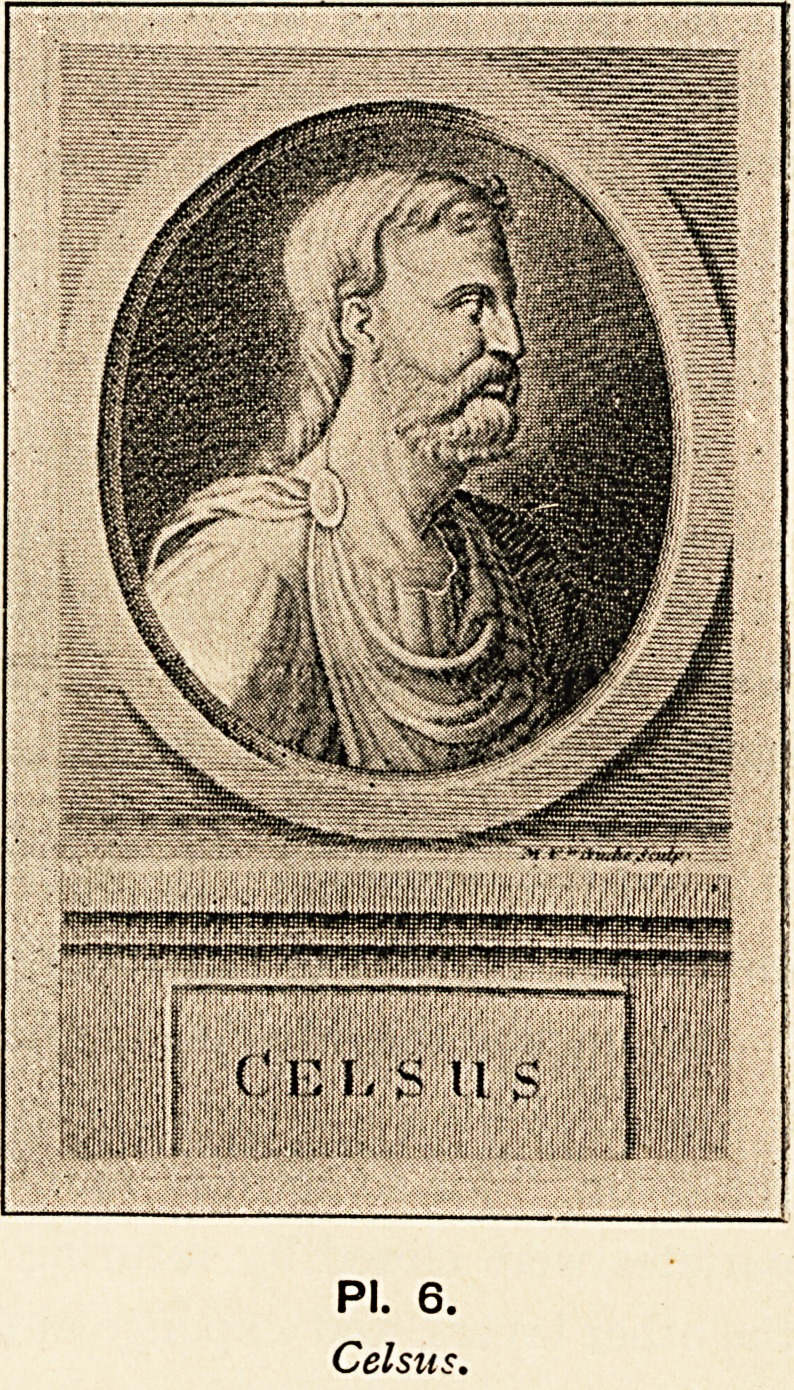


**Pl. 7. f7:**
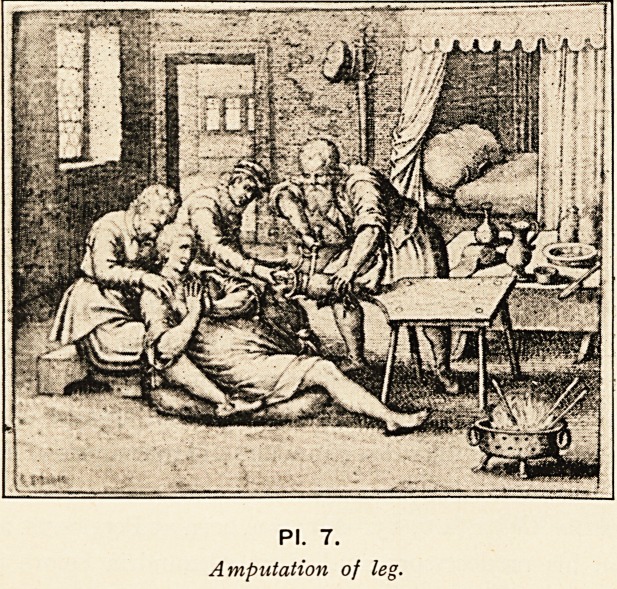


**Pl. 8. f8:**
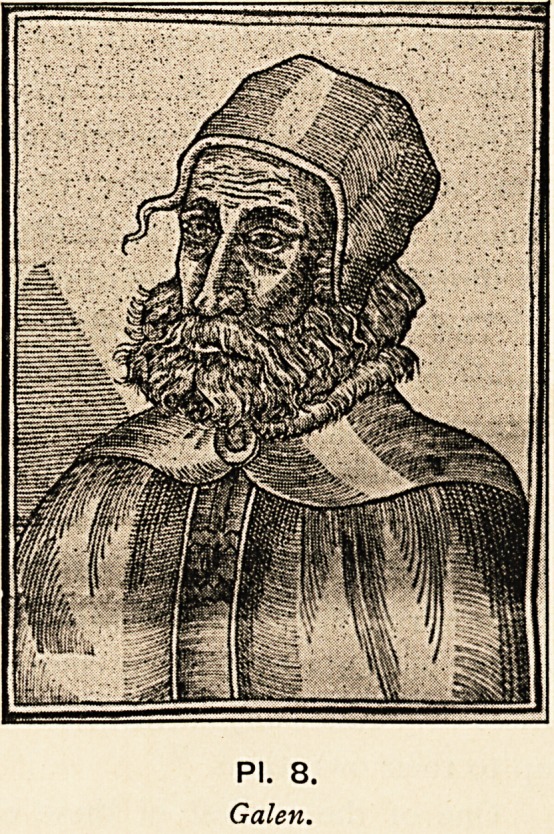


**Pl. 9. f9:**
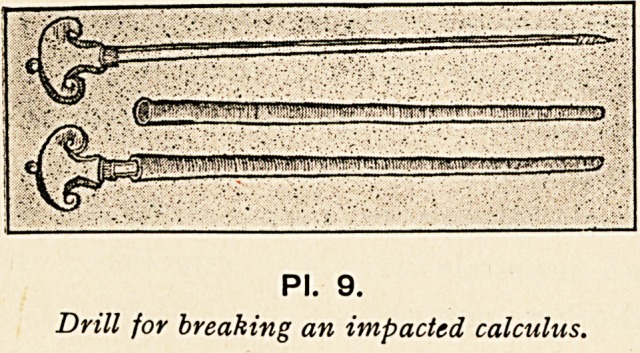


**Pl. 10. f10:**